# Identification of MicroRNAs in the Coral *Stylophora pistillata*

**DOI:** 10.1371/journal.pone.0091101

**Published:** 2014-03-21

**Authors:** Yi Jin Liew, Manuel Aranda, Adrian Carr, Sebastian Baumgarten, Didier Zoccola, Sylvie Tambutté, Denis Allemand, Gos Micklem, Christian R. Voolstra

**Affiliations:** 1 Red Sea Research Center, King Abdullah University of Science and Technology (KAUST), Thuwal, Saudi Arabia; 2 Cambridge Systems Biology Centre & Department of Genetics, University of Cambridge, Cambridge, United Kingdom; 3 Centre Scientifique de Monaco, Monaco, Monaco; Pennsylvania State University, United States of America

## Abstract

Coral reefs are major contributors to marine biodiversity. However, they are in rapid decline due to global environmental changes such as rising sea surface temperatures, ocean acidification, and pollution. Genomic and transcriptomic analyses have broadened our understanding of coral biology, but a study of the microRNA (miRNA) repertoire of corals is missing. miRNAs constitute a class of small non-coding RNAs of ∼22 nt in size that play crucial roles in development, metabolism, and stress response in plants and animals alike. In this study, we examined the coral *Stylophora pistillata* for the presence of miRNAs and the corresponding core protein machinery required for their processing and function. Based on small RNA sequencing, we present evidence for 31 *bona fide* microRNAs, 5 of which (miR-100, miR-2022, miR-2023, miR-2030, and miR-2036) are conserved in other metazoans. Homologues of Argonaute, Piwi, Dicer, Drosha, Pasha, and HEN1 were identified in the transcriptome of *S. pistillata* based on strong sequence conservation with known RNAi proteins, with additional support derived from phylogenetic trees. Examination of putative miRNA gene targets indicates potential roles in development, metabolism, immunity, and biomineralisation for several of the microRNAs. Here, we present first evidence of a functional RNAi machinery and five conserved miRNAs in *S. pistillata*, implying that miRNAs play a role in organismal biology of scleractinian corals. Analysis of predicted miRNA target genes in *S. pistillata* suggests potential roles of miRNAs in symbiosis and coral calcification. Given the importance of miRNAs in regulating gene expression in other metazoans, further expression analyses of small non-coding RNAs in transcriptional studies of corals should be informative about miRNA-affected processes and pathways.

## Introduction

Scientific curiosity about corals has recently intensified, following observations of the deterioration of coral reefs at an unprecedented rate worldwide — for instance, in the Caribbean, Hughes [1] reported that coral cover has declined from over 50% in the 1970s to less than 5% in the 1990s; in the Indo-Pacific region, home to 75% of the world's coral reefs, Bruno and Selig [2] estimated that coral cover declined ∼1% annually in the past 20 years, and ∼2% annually between 1997–2003. This trend is worrying, as coral reefs are important ecosystems, supporting more marine biodiversity per unit area than any other marine habitat [3]. There are many reasons behind the global decline of coral reefs, which include, but are not limited to, accelerated warming and acidification of oceans [4], [5], overfishing [1], pollution [6], [7], and disease [8].

In recent years, the increasing use of genomics has broadened our understanding of basic coral biology. The genome sequence of the coral *Acropora digitifera* [9] revealed a potential dependency of some coral species on their symbiont population for synthesis of an essential amino acid, and highlighted an unexpectedly diverse repertoire of immune-response genes [9]. Furthermore, microarray and RNA sequencing studies on several coral species have shed light on their responses to environmental cues at the transcriptional level. Shifts in transcriptional landscapes have been noted, based on the composition of symbionts in the coral cell [10], [11], or as a response to stressors such as increased temperatures [12]–[15]; long-term darkness [16]; elevated CO_2_ levels [17], [18], and ultraviolet radiation [19]. Despite the increasing accumulation of genomic data, some aspects of the molecular machinery potentially involved in these processes, such as microRNAs (miRNAs), have yet to be studied in corals.

miRNAs are a class of small non-coding RNAs of ∼22 nucleotides (nt) in length, which regulate gene expression through posttranscriptional degradation or translational repression via the RNA interference pathway (RNAi) [20]–[22]. Recent studies in plants and metazoans have discovered pivotal roles for miRNAs in regulating developmental timing [23]–[25]; cell cycle progression [26], [27]; immune response [28], [29]; metabolism [30]; response to stress [31]–[33]; and potentially biomineralisation [34]–[36]. miRNAs have been identified in more than 200 species that span major kingdoms of life: animals, plants, and protists (based on miRBase v20, June 2013) [37]–[40]. miRNAs have also been identified in the genome and transcriptome of the coral symbiont *Symbiodinium microadriaticum* [41] as well as in the genomes of two other cnidarians: Chapman et al. [42] reported 17 miRNAs for *Hydra magnipapillata*, while Grimson et al. [43] reported 40 miRNAs in the sea anemone *Nematostella vectensis*. The large evolutionary distance from *Hydra* and *Nematostella* to corals (∼500 million years [9]) warranted a search for the presence of miRNAs and the corresponding RNAi machinery in scleractinian corals. Here we present a first assessment of the miRNA repertoire, the RNAi machinery, and putative gene targets in the scleractinian coral *S. pistillata* from the Red Sea.

## Materials and Methods

### Ethics statement

Corals were kept in accordance with recommendations by the Centre Scientifique de Monaco and appropriate guidelines for the Care and Use of Laboratory Animals (Permit Number DCI/89/32).

### Growth conditions of *S. pistillata*

*S. pistillata* was maintained in aquaria at the Centre Scientifique de Monaco, Principality of Monaco, in controlled culture conditions: semi-open circuit, Mediterranean seawater heated to 25±0.5°C, salinity of 38.2 psu, illuminated with HQI-10000K; BLV-Nepturion at a constant irradiance of 175 µmol photons m^−2^ s^−1^ on a 12 h∶12 h day∶night light cycle. Corals were fed three times a week with a mix of *Artemia salina nauplii* and *A. salina* frozen adults and frozen krill.

### mRNA sequencing and transcriptome

Total RNA extraction was performed as described previously [44]. Briefly, nubbins of coral (sampled at noon) were snap-frozen in liquid nitrogen and ground into powder in a cryogrinder (Freezer/Mill 6770, Spex Sample Prep) and then extracted with TRIzol Reagent (Invitrogen, Carlsbad, CA) according to manufacturer's instructions. Total RNA was quality-checked using a Bioanalyzer 2100 (Agilent, Santa Clara, CA) and a Nanodrop 2000c (ThermoScientific, Wilmington, DE) prior to library creation and sequencing by the KAUST Bioscience Core lab. For mRNA sequencing, paired-end reads for Illumina sequencing were generated from oligo-dT selected total RNA using the Illumina TruSeq RNA Sample Prep Kit (Illumina, San Diego, CA) according to manufacturer's instructions. A total of 152,552,099 paired-end read pairs (read length: 101 bp, insert size: 175 bp) were sequenced on the HiSeq 2000 platform (Illumina, San Diego, CA).

For the transcriptome assembly, sequence adaptors were trimmed from the raw sequences and low quality ends were cut with trimmomatic [45]. The remaining read pairs were subjected to digital normalization with Diginorm at k = 20 and C = 20 [46], reducing the dataset to 51,023,864 read pairs. Further, in order to remove contaminating sequence information from endosymbiotic dinoflagellates, remaining read pairs were mapped to the transcriptome of *Symbiodinium microadriaticum* [41] using Bowtie 2 [47]. This resulted in 38% of the remaining read pairs being mapped to the *S. microadriaticum* transcriptome, and a significant reduction in potential chimeric locus assemblies for the remaining 16,555,086 read pairs.

The transcriptome was assembled with Oases [48] using k-mer values ranging from 29 to 69. To reduce redundancy within single k-mer assemblies, only contigs with a minimum coverage of 7 were reported. Based on contig lengths, number of distinct loci, and number of transcripts, single k-mer assemblies from k = 45, 47, 49 were reassembled at k = 27, resulting in a final transcriptome assembly of 43,493 unique loci/genes ≥250 bp (Supporting Information S1). For gene annotation, the longest transcript per loci was subjected to a BLASTX search (minimum e-value threshold of 10^−5^) against three protein databases: UniProtKB/Swiss-Prot, UniProtKB/TrEMBL, and the non-redundant GenBank nr in a subsequent manner. Hits were selected preferentially from Swiss-Prot as Swiss-Prot is manually curated, followed by TrEMBL if no matches were found in Swiss-Prot, and lastly from nr if neither Swiss-Prot nor TrEMBL yielded hits. Out of the 20,332 transcripts with an annotation, 15,177 (77.6%) were from Swiss-Prot; 4,964 (24.4%) were from TrEMBL; and 193 (0.93%) were from nr (Supporting Information S2). For transcripts with annotations from Swiss-Prot or TrEMBL, a script was written to assign GO (Gene Ontology) terms (and their parent GO terms) from UniProt-GOA [49]. 14,558 (95.9%) of the Swiss-Prot hits and 1,955 (39.4%) of the TrEMBL hits had at least 1 GO term assigned to it (Supporting Information S2).

### Identification of core RNAi proteins

In order to identify homologues of the RNAi machinery in *S. pistillata*, sequences from six families of proteins (Argonaute, Dicer, Piwi, Drosha, Pasha, and HEN1) were drawn from five organisms (*H. sapiens*, *D. melanogaster*, *C. elegans*, *S. pombe*, and *A. thaliana*). These sequences were obtained from the UniRef100 database [50], and clustered into groups with 90% sequence identity using CD-HIT [51] to remove near-identical sequences. The clustered sequences were used in a TBLASTN search against the *S. pistillata* transcriptome to identify candidate RNAi-related transcripts. Identified homologues (TBLASTN e-value<10^−10^) of known RNAi proteins were then searched for domains that are required for the function of the protein using InterProScan [52]–[54]. The domains that were determined to be essential for function were: a Paz and Piwi domain for Argonaute and Piwi; a pair of RNase III domains for Dicer and Drosha; a double-stranded RNA binding domain for Pasha; and a methyltransferase (MTase) domain for HEN1. Candidate homologues were not considered further in the absence of any of these domains.

Additional support for the inferred function of candidate homologues was obtained by carrying out a reciprocal BLASTP search of these translated candidates against all proteins in the Swiss-Prot database [55] (Supporting Information S3). The candidate homologues were aligned against known RNAi proteins on a per-family basis using Clustal Omega [56], and the alignments were visualised using Jalview [57], [58]. Key residues were derived from literature [59]–[64]. In addition, for each of the six protein families, phylogenies were constructed by aligning our candidate homologues with selected sequences from Grimson et al. [43] and Moran et al. [65] with MUSCLE [66]. Aligned regions of low quality were removed with TrimAl, using the in-built “gappyout” parameter [67] (Supporting Information S4). ProtTest3 [68] was used to determine the best model for amino acid substitution, and MEGA (version 6) [69] was used to construct maximum-likelihood phylogenetic trees (support values were computed from 1,000 bootstrap replicates).

### Small RNA sequencing and processing

The small RNA (smRNA) fraction from *S. pistillata* was selectively enriched from isolated total RNA (see above) using the mirVana miRNA isolation kit (Ambion, Austin, TX) according to manufacturer's instructions. The small RNA fraction was quality-checked using a Bioanalyzer 2100 (Agilent, Santa Clara, CA) and a Nanodrop 2000c (ThermoScientific, Wilmington, DE). The small RNA library was created using the Illumina Small RNA Sample Prep Kit (Illumina, San Diego, CA) according to manufacturer's instructions, and sequenced on 1 lane on an Illumina Genome Analyzer IIx (GA2x) machine. A total of 30.5 million small RNA reads of <40 bp in length were produced. The reads, along with associated Phred quality scores for each sequenced base, were saved in a FASTQ file.

The raw FASTQ file was processed using several scripts to remove low-quality reads resulting in a more compact FASTQ file that contained high-quality reads for downstream analyses. First, low quality 3′ ends were trimmed from the reads. The 3′ end of the resulting reads had a Phred score >20, while the average Phred score of the entire read was >20 as well. After trimming, the overall error rate of the reads was calculated from the Phred scores of individual bases. Reads were discarded if the error rate exceeded 2%. Subsequently, the Illumina 5′ and 3′ adapter sequences used in library generation were trimmed off using Cutadapt v1.0 [70]. Last, in order to remove fragments of rRNA, tRNA, and mRNA sequences, Velvet [71] was used to assemble the short reads into contigs (at k = 25), which were then compared to the GenBank nt database (nucleotide collection at NCBI). In addition, we compared the assembled contigs to the *S. pistillata* transcriptome assembly using BLASTN, in order to remove short reads that matched known mRNA sequences.

### miRNA prediction and filtering

We used miRDeep2 [72] to identify miRNAs. Briefly, miRDeep2 mapped smRNAs to a preliminary draft genome of *S. pistillata* using Bowtie, discarding reads that occurred more than five times in the genome to avoid mapping to repetitive elements. Potential pre-miRNA precursor sequences were identified based on the pattern of the mappings, and subsequently folded using RNAfold to ascertain whether they had the canonical hairpin secondary structure [72]. Predicted pre-miRNAs that had a miRDeep2 score of 10 or above were retained for further analyses and inspected manually. A script was written to produce additional information not found in the miRDeep2 output (i.e. length of 3′ overhang, proportion of reads with consistent 5′ end, number of mismatched bases in stem) to further select a set of *bona fide* miRNAs. Conserved miRNAs were identified using BLASTN against all previously identified pre-miRNA sequences in miRBase (ver 20) [37]–[40].

### Functional analysis of miRNA targets

ORFs were identified in the transcripts using TransDecoder (part of the Trinity software pipeline [73]). Sequences downstream of the longest ORF identified in the transcripts were treated as the 3′ UTR of the transcript. 3′ UTRs under 100 bp were filtered out to remove transcripts associated with short UTR sequences. Out of the 43,493 genes, 14,125 transcripts (32.4%) had a predicted 3′ UTR longer than 100 bp.

For each miRNA, we ran PITA (Probability of Interaction by Target Accessibility) [74] on the 3′ UTRs at default settings to produce a set of putative genes targeted by the miRNA. In the absence of genomic data from other closely related organisms, PITA achieves higher sensitivity and specificity than other target prediction software (e.g. miRanda, TargetScan) as the latter algorithms rely on a filter based on evolutionary conservation to reduce the false positive rate. PITA works by calculating the difference in Gibbs' free energy (ΔΔG) between the energy that is required to unfold the secondary structure of the target site (ΔG_open_), and the energy of the mature miRNA binding its target (ΔG_duplex_) [74]. Only miRNA targets with a ΔΔG of ≤−10 kcal mol^−1^ were retained.

For GO enrichment of target genes, we used topGO (version 2.12.0), an R script that is available through Bioconductor 2.0. topGO is a scoring algorithm that improves GO scoring by eliminating local dependencies between related GO terms [75]. The threshold for significance was set at P<0.01, using otherwise default topGO “weight01” settings, which produced GO terms that were significantly enriched in the set of transcripts targeted by each miRNA. The resulting P values were not corrected for multiple testing, as non-independent tests are carried out on each GO term by topGO [75].

## Results

### Identification of core RNAi proteins

The miRNA machinery that processes and mediates the function of miRNAs encompasses several key components that appear to be conserved across the animal kingdom [76]. In order to establish the presence of a functional miRNA machinery in *S. pistillata* we conducted a BLAST-based search for key proteins known to be essential for miRNA processing and function.

We identified seven candidate genes that are homologues to known RNAi proteins: one Argonaute, two Piwi, one Dicer, one Drosha, one Pasha, and one HEN1 in *S. pistillata*. We employed several key metrics (i.e. matches to known RNAi families, presence of protein domains crucial for catalytic activity, and a reciprocal BLAST search against manually curated proteins in Swiss-Prot) to identify candidate RNAi proteins (Supporting Information S3).

**Figure 1:**
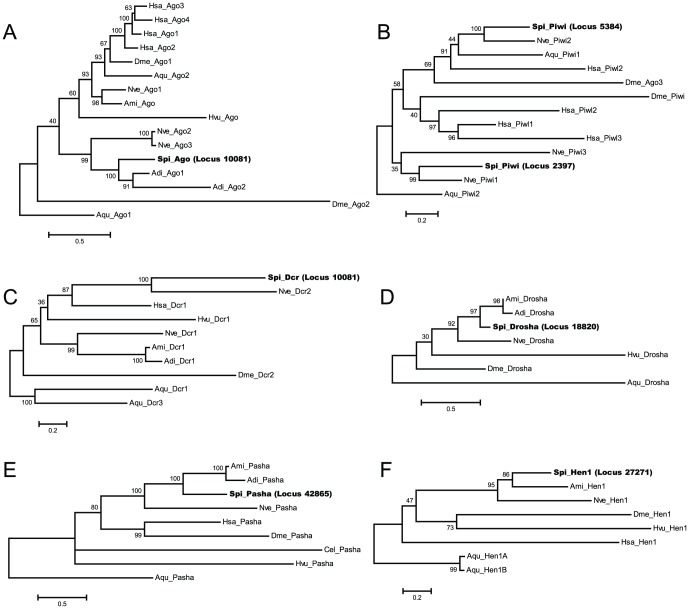
Maximum-likelihood phylogenies of (A) Argonaute, (B) Piwi, (C) Dicer, (D) Drosha, (E) Pasha, and (F) HEN1 proteins. (A), (C), and (D) were constructed using the amino acid substitution model LG+G, while (B), (E), and (F) were constructed using LG+I+G. Bootstrap support values are indicated above the branches. Abbreviations used in these trees are ‘Adi’: *Acropora digitifera*; ‘Ami’: *Acropora millepora*; ‘Aqu’: *Amphimedon queenslandica* (a sponge); ‘Cel’: *C. elegans*; ‘Dme’: *D. melanogaster*; ‘Hsa’: *H. sapiens*; ‘Hvu’: *Hydra vulgaris*; ‘Nve’: *N. vectensis*; and ‘Spi’: *S. pistillata*.

The per-family alignments of candidate homologues against known sequences revealed a striking conservation of functionally important amino acid residues located within the key protein domains. Examples include the strong conservation of the DDX triad in the Piwi domain of the Argonaute and Piwi homologues; the aspartate and glutamate residues essential for Dicer activity; and the pair of alanine/alanine and alanine/serine dipeptides involved in the binding of dsRNA in Pasha (Supporting Information S5, S6, S7, S8, S9, S10). Maximum-likelihood phylogenetic trees that were constructed for all six protein families (Figures 1A–1F) placed all of the candidate *S. pistillata* homologues with those from other cnidarians. Judging from the presence of the key RNAi proteins in *S. pistillata* in comparison to other organisms, the RNAi machinery in *S. pistillata* is similar in composition to those from sea anemone, worm, fruit fly, and humans (Table 1).

**Table 1:** Presence of RNAi proteins in *S. pistillata* in comparison to Cnidaria and other model organisms (‘+’: presence, ‘−’: absence, ‘?’: not determined).

	Organism	Ago	Piwi	Dicer	Drosha	Pasha	HEN1
**Cnidaria**	*S. pistillata*	+	+	+	+	+	+
	*N. vectensis*	+	+	+	+	+	+
	*A. millepora*	+	?	+	+	+	+
	*A. digitifera*	+	?	+	+	+	−
	*H. vulgaris*	+	?	+	+	+	+
	*H. magnipapillata*	+	+	+	+	?	?
**Other**	*H. sapiens*	+	+	+	+	+	+
	*D. melanogaster*	+	+	+	+	+	+
	*C. elegans*	+	+	+	+	+	+
	*A. thaliana*	+	−	+	−	−	+
	*S. pombe*	+	−	+	−	−	−

Besides the core RNAi proteins, we have also discovered transcripts that are candidate homologues of HYL1 (one), GW182 (two), and RdRP (RNA-dependent RNA polymerase, eight) (data not shown). HYL1 is thought to be a plant-specific partner to Dicer [77], whereas GW182 helps Argonaute repress its targets [78]. Both proteins have recently been discovered in four cnidarians (*Acropora digitifera*, *A. millepora*, *Hydra vulgaris*, *Nematostella vectensis*). However, although we could identify the PAM2 and P-GL motif in one of our GW182 homologues, there were very few GW repeats in this homologue (1 in *S. pistillata*, compared to 14 in *N. vectensis* and 40 in humans) [65]. RdRPs, using small RNAs as templates, amplify the silencing effect by directing the production of secondary dsRNAs [79]. Functional RdRPs have been discovered in plants [25], [80] and *C. elegans* [81], but not in mammals nor flies [79]. Four candidate homologues of RdRPs have been found in *N. vectensis* [82], indicating that RdRPs might be present in cnidarians.

### Small RNA sequencing and miRNA repertoire

**Figure 2:**
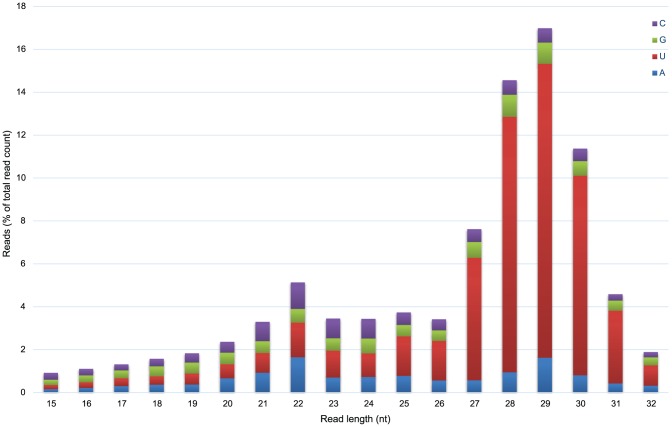
Frequency distribution of small RNA reads in the *S. pistillata* sequencing library. The bars are coloured to reflect the proportions of reads starting with A, U, G, and C (blue, red, green, and purple respectively).

Sequencing produced 30,543,433 reads, of which 23,830,932 reads (78.0%) were kept after adapter trimming. The additional step of removing short reads that matched known rRNA, tRNA, and transcript sequences removed a further 7.2% of reads, resulting in a dataset that contained 21,625,195 reads of at least 10 bp in length. Most of these reads were of 20–31 nt in length. Relative frequencies of the starting 5′ nucleotide showed a clear enrichment of 5′-terminal uracil among short reads of length 26–31 nt (Figure 2), which is consistent with the likely presence of functional Piwi-interacting RNAs (piRNAs) in *S. pistillata* [43].

A total of 2,811,736 reads (of length >17 nt) were mapped to a preliminary assembly of the *S. pistillata* genome and 46 distinct miRNAs were predicted by miRDeep2, of which a subset of 30 miRNAs passed additional filtering criteria (see Materials and Methods). An exception was made for spi-miR-temp-25 – although the precursor had a 3 bp 3′ overhang, it was included in the *bona fide* set as it was a close match to two known miRNAs. The resulting 31 miRNAs were used in all downstream analyses (Table 2, Supporting Information S11).

**Table 2:** Set of 31 *bona fide* miRDeep2-predicted miRNAs in *S. pistillata*.

miRNA name^1^	Predicted mature miRNA (5′ – 3′)	Matches to known miRNAs
spi-miR-temp-1	acccguagauccgaacuugugg	miR-100 family
spi-miR-temp-2	uaucgaauccgucaaaaagaga	
spi-miR-temp-3	ucagggauuguggugaguuaguu	
spi-miR-temp-4	aaagaaguacaagugguaggg	nve-miR-2023
spi-miR-temp-5	gagguccggaugguuga	
spi-miR-temp-6	uaucgauuccgucaaaaagaga	
spi-miR-temp-7	uaugauaucguauccuuugagg	
spi-miR-temp-8	aaguuugagauuugauuuacugaag	
spi-miR-temp-9	ucucugaaaucuccuaagcuauca	
spi-miR-temp-10	ucaguuccaccaucucaccuaua	
spi-miR-temp-12	ggaguuuguuguacugugcuauu	
spi-miR-temp-13	ugggauuaaaacuucuucggugugg	
spi-miR-temp-14	caauguuucggcuuguucccg	
spi-miR-temp-15	ucaagucuaggcugguuaguuu	
spi-miR-temp-16	uuuaguuuuccgauauuuuuagg	
spi-miR-temp-17	ugaacccagaaccucgaagg	
spi-miR-temp-18	ugaaauacucugacggagucagu	
spi-miR-temp-19	ugucauauccauccaaacgagg	
spi-miR-temp-20	ugugauuggagacuuuuaucgu	
spi-miR-temp-22	ccgauuugaacaauguuccguuc	
spi-miR-temp-23	aaauugcuccgaaauacaucuau	
spi-miR-temp-25	uuugcuaguugcuuuugucccguu	nve-miR-2022 and hma-miR-2022
spi-miR-temp-26	uccagcaccaauguuauuguua	
spi-miR-temp-29	uggcauaagggcagccaccccuu	
spi-miR-temp-30	uauauuguacgacucucaucgugu	nve-miR-2036
spi-miR-temp-33	ccaacugugacugcaaauuaau	
spi-miR-temp-34	acugauauucaccaagugauua	
spi-miR-temp-36	gaaaaguucgucgaucacucg	
spi-miR-temp-38	ucaguuccaccaucucaccuac	
spi-miR-temp-40	uagcauaacauuguaagagauc	nve-miR-2030
spi-miR-temp-42	ugugcaagaauuugagucgcugg	

1 Note: the names of the miRNAs are temporary. miRBase (the miRNA registry) only accepts submissions of new miRNAs after the manuscript has been accepted for publication.

**Figure 3:**
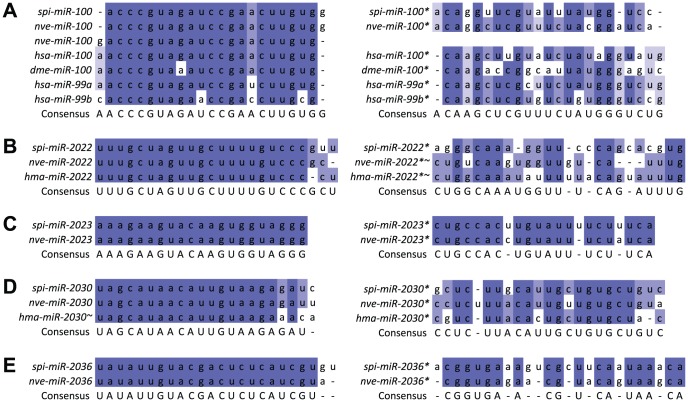
Alignments of predicted *S. pistillata* miRNAs against (A) members of the miR-100 family; (B) nve- and hma-miR-2022; (C) nve-miR-2023; (D) nve-miR-2030; and (E) nve-miR-2036. The mature sequences are shown on the left, while star sequences are on the right. Sequences were obtained from miRBase (version 20). The mature hma-miR-2030 aligned best with miR-2030* sequences from *N. vectensis* and *S. pistillata*. Sequences marked with a tilde (nve-miR-2022*, hma-miR-2022*, and hma-miR-2030) are miRNAs that we derived based on the alignment of the respective pre-miRNA sequences obtained from miRBase against *S. pistillata* miRNAs. Bases were coloured to provide visual indication of conservation (dark blue: >80%; blue: >60%; light blue: >40%; uncoloured otherwise). Abbreviations used are ‘dme’: *D. melanogaster*; ‘hma’: *H. magnipapillata*; ‘hsa’: *H. sapiens*; ‘nve’: *N. vectensis*; and ‘spi’: *S. pistillata*.

While the majority of these 31 predicted miRNAs were novel, 5 of them matched conserved miRNAs. spi-miR-temp-1, the predicted miRNA with the highest miRDeep2 score, was highly similar to the known miR-100 family of sequences (∼2 mismatched bases, Figure 3A). This family is known to be conserved across Eumetazoa, including at least two other cnidarians (*N. vectensis* and *Metridium senile*) [43], [83]–[87]. spi-miR-temp-25 was similar to miR-2022 from *N. vectensis* [43] and *H. magnipapillata* [88] (Figure 3B), while the other three miRNAs – spi-miR-temp-4, spi-miR-temp-40, and spi-miR-temp-30 – were similar to miR-2023, miR-2030, and miR-2036 found in *N. vectensis* [43], respectively (Figures 3C–E). For reasons of clarity, these five conserved miRNAs in *S. pistillata* will be referred to as spi-miR-100, spi-miR-2022, spi-miR-2023, spi-miR-2030, and spi-miR-2036, in accordance with miRNA naming conventions.

Although nve-miR-100 has been identified in two separate studies (both of which utilised next-generation sequencing of short reads to identify miRNAs in basal metazoans), Grimson et al. [43] and Wheeler et al. [88] predicted mature miR-100 sequences which are offset by a single nucleotide. From our read data, we had >30,000 reads that exactly match the nve-miR-100 from Grimson et al. [43], but none that matched the alternative version from Wheeler et al. [88]. A negligible minority of reads (∼10) did start one nucleotide upstream, but unlike the Wheeler et al. [88] version, this residue was an adenine, making this form identical to the hsa-, dme- and xtr-miR-100s.

### Functional analysis of putative miRNA targets

In order to identify processes in *S. pistillata* that are potentially regulated by miRNAs, we conducted a GO term enrichment analysis. Briefly, for the set of 31 miRNAs, we searched for animal-like target sites in the 3′ UTRs of those 16,513 *S. pistillata* genes that had available 3′ UTR and GO annotation. This analysis was performed for all miRNAs individually, and indicated that miRNAs are likely to play roles in diverse processes (Table 3, Supporting Information S12).

**Table 3:** Enriched immunity- and biomineralisation-related GO terms (default topGO settings, P<0.01) associated with predicted miRNA target genes from *S. pistillata*.

miRNA	GO ID	Description	P value
***Immunity***			
spi-miR-2022	GO:0044003	modification by symbiont of host morphology or physiology	0.0015
spi-miR-2022	GO:0019048	virus-host interaction	0.0086
spi-miR-temp-15	GO:0044130	negative regulation of growth of symbiont in host	0.00634
spi-miR-temp-15	GO:0032735	positive regulation of interleukin2 production	0.00119
spi-miR-temp-15	GO:0034134	toll-like receptor 2 signaling pathway	0.00188
spi-miR-temp-15	GO:0034142	toll-like receptor 4 signaling pathway	0.00626
spi-miR-temp-15	GO:0038123	toll-like receptor TLR1:TLR2 signaling pathway	0.00314
spi-miR-temp-15	GO:0038124	toll-like receptor TLR6:TLR2 signaling pathway	0.00314
spi-miR-temp-16	GO:0070498	interleukin-mediated signaling pathway	0.00019
spi-miR-temp-16	GO:0042102	positive regulation of T cell proliferation	0.00366
spi-miR-temp-20	GO:0032088	negative regulation of NF-kappaB transcription factor activity	0.0038
spi-miR-temp-26	GO:0051703	intraspecies interaction between organisms	0.0086
***Biomineralisation***			
spi-miR-100	GO:0060348	bone development	0.00475
spi-miR-temp-7	GO:0030574	collagen catabolic process	0.00733
spi-miR-temp-8	GO:0001958	endochondral ossification	0.0069
spi-miR-temp-12	GO:0003417	growth plate cartilage development	0.00891
spi-miR-temp-13	GO:0070588	calcium ion transmembrane transport	0.0046
spi-miR-temp-15	GO:0060346	bone trabecula formation	0.00629
spi-miR-temp-15	GO:0003417	growth plate cartilage development	0.00119
spi-miR-temp-15	GO:0043931	ossification involved in bone maturation	6.50E-05
spi-miR-temp-16	GO:0045672	positive regulation of osteoclast differentiation	0.00776
spi-miR-temp-18	GO:0030509	BMP signaling pathway	0.0084
spi-miR-temp-18	GO:0030501	positive regulation of bone mineralization	0.0031
spi-miR-temp-19	GO:0015701	bicarbonate transport	0.00172
spi-miR-temp-23	GO:0061036	positive regulation of cartilage development	0.00066

We categorised the resulting enriched GO terms under several high-level groups based on known miRNA function – “immunity”, “biomineralisation”, “transcription”, “cell cycle”, “cytoskeleton”, “metabolism”, “transport/signalling”, and “differentiation/development”. Other GO terms that did not fall under one of these umbrella terms were categorised under “miscellaneous” (Supporting Information S12).

We paid particular attention to the “immunity” and “biomineralisation” high-level groups, as we considered these terms to be specifically relevant to the understanding of coral physiology (Table 3). For the former, it is likely that immunity-related transcripts are involved in the formation and retention of symbiotic relationships between the coral host and its *Symbiodinium* symbionts. Five miRNAs were predicted to be involved in this regulation, with a large fraction of the predictions involving spi-miR-temp-15. For the latter, 10 miRNAs were predicted to be involved in the formation of the calcium carbonate-based coral skeleton, with none of the 10 miRNAs being predominant in the predictions.

## Discussion

### Identification of core RNAi proteins

Advances in our understanding of miRNAs have shown that these small molecules have a big impact on the regulation of gene expression. While the biogenesis and downstream functions of miRNAs have been fairly well studied in the primary model organisms, little is known about the presence or function of these miRNAs in corals. In this study, we identified the presence of core RNAi proteins encoded by the *S. pistillata* transcriptome. The alignment of our candidate homologues against known RNAi proteins revealed the conservation of key protein domains and residues crucial for protein function. Additionally, maximum-likelihood phylogenetic trees of our candidate homologues with RNAi proteins from other cnidarian and model organisms showed broad agreement with those from other studies [43], [65] – all of our seven candidate homologues clustered best with those from other cnidarians, as expected from its closer phylogenetic distance to other cnidarians than to bilaterians or poriferans. This also signifies that our homologues originate from the coral host, not from its dinoflagellate symbionts. Interestingly, the *S. pistillata* Dicer homologue clustered better with *N. vectensis* Dcr2, which is thought to be involved in processing of long dsRNA into siRNAs, and not associated with the biogenesis of miRNAs [65], [89]. A reverse search of our candidate Dicer homologue against the *S. pistillata* draft genome revealed an open reading frame that encodes for another Dicer-like protein, which appears to be a good match (e-value of <1×10^−10^) of *N. vectensis* Dcr1 (data not shown). However, the absence of transcriptomic support for that open reading frame excluded it from being a candidate Dicer in *S. pistillata* in this study. Nonetheless, both observations serve to indicate the presence of a functional miRNA-processing machinery in *S. pistillata*. This, to our knowledge, has not been demonstrated previously for any other coral.

### Small RNA sequencing and miRNA repertoire

Besides a functional RNAi machinery, and based on our analysis of short reads, we also predicted the presence of 31 *bona fide* miRNAs (out of a total of 46), of which 5 were conserved: the miR-100 family found in many other metazoans; miR-2022, which is conserved in *N. vectensis* and *H. magnipapillata*; miR-2023, miR-2030, and miR-2036, which are conserved in *N. vectensis* only. The dearth of conserved Hydra miRNAs in *S. pistillata* echoes the findings of Chapman et al. [42], [43], who found only one conserved *N. vectensis* miRNA among the *H. magnipapillata* miRNAs. This might be due to the evolutionary distance separating the anthozoans and hydrozoans, or, more likely, due to the incomplete coverage of short reads used in the identification of miRNAs in *H. magnipapillata* – only 9,654 reads were used to identify potential miRNA genes in *H. magnipapillata* [42]. In contrast, we (and Grimson et al. [43]) identified miRNAs from a much larger pool of short reads. We believe that the repertoire of miRNAs that are conserved across both cnidarian classes (i.e. Anthozoa and Hydrozoa) could be expanded if miRNA predictions were ran on a larger pool of small RNA reads.

The conservation of miRNA families across and within different bilaterian phyla have been fairly well-covered, with the general consensus that the continuous acquisition of miRNA families with minimal secondary losses rapidly expanded the bilaterian miRNA repertoire relative to cnidarians, which contributes to the increased morphological complexity of bilaterians [83], [88], [90]–[92]. As one of the few cnidarians with its small RNA fraction extensively sequenced, *S. pistillata* has demonstrated that conservation of miRNA families does occur within cnidarians too, as five of its miRNAs are conserved in *N. vectensis* despite the ∼500 mya evolutionary distance that separate both species. However, due to the dearth of sequenced small RNA reads from other cnidarians, we are unable to make further conclusions regarding the rate at which cnidarians acquire their own phylum-specific miRNA families. Also, recent evidence has surfaced that demonstrated the gradual loss of conserved (up to 50% in more derived species) and gain of novel miRNA families in Platyhelminthes, the first that was reported for a major lineage within Bilateria, and might be related to morphological simplifications in some of the studied flatworms [93]. Similar observations could apply to specific classes of cnidarians, but this type of study would need to include more than just a few species of cnidarians in order to elucidate the true rate underlying the gains and losses of miRNA families.

### Functional analysis of putative miRNA targets

Functional analysis of all 31 miRNAs, using target predictions for each miRNA followed by a GO enrichment analysis on the predicted target genes, revealed several putative processes and pathways that are regulated by miRNAs in corals. For the miR-100 homologue in *S. pistillata*, the GO terms “embryonic forelimb morphogenesis” and “bone development” were enriched (P<0.01, Supporting Information S12) in the predicted targets, which is reminiscent of its reported function: in humans, miR-100 has been shown to target genes involved in growth and development. Examples include Plk1, a key mitotic checkpoint regulatory protein [26]; RBSP3, involved in cell proliferation and myeloid cell differentiation [27]; BMPR2, involved in osteogenesis [94]; and FRAP1/mTOR, which regulates cell growth [95]. It is possible that miR-100 plays an analogous role in coral calcification, making this miRNA a potentially important piece of the puzzle in coral physiology, as well as a gene of interest when investigating coral responses to ocean acidification. However, as miRNA-mRNA target recognition depends critically on the miRNA seed sequence (bases 2–7 of the mature RNA), it is possible that the targets of bilaterian and cnidarian miR-100 will differ due to the one nucleotide offset between the two miRNA sequences. This 5′ offset has also been observed for miR-2, miR-10, miR-133, and miR-210 that are otherwise well-conserved across two phylogenetically-related taxa, and presumably able to regulate non-overlapping sets of target mRNAs [91]. Thus, further experimentation is required to confirm the *bona fide* function of cnidarian miR-100 in corals. Nonetheless, our spi-miR-100 adds to the existing literature documenting the strong conservation of miR-100 within metazoans.

Besides the only miRNA with documented function, we identified miRNAs whose targets are involved in high-level functions such as immunity, biomineralisation, regulation of cell cycle, cellular motility, metabolism, signalling, and development, analogous to functions that were previously ascribed to miRNAs in other organisms [23]–[36]. We were interested in the first two high-level groups, as immunity genes might regulate the relationship with the symbiotic dinoflagellate *Symbiodinium*, and biomineralisation genes may control the rate of coral skeleton growth, two processes that are arguably of importance to corals under conditions of environmental change.

Out of the 5 miRNAs that were predicted to regulate coral immunity genes, we speculate that spi-miR-temp-15 should warrant further investigation due to the significant enrichment of multiple immunity-related GO terms in the transcripts targeted by this miRNA. Indeed, several of the predicted target genes of spi-miR-temp-15 have homologues that are known to be regulated by other miRNAs: Nod2 is repressed by miR-122 [96]; TLR2 is regulated by miR-19 and miR-105 [97], [98]; while caspase-8 is targeted by miR-874 [99]. Interestingly, this miRNA is not conserved in *N. vectensis*, which does not form long-term symbiotic relationships with *Symbiodinium*.

In contrast to the previous category, 10 miRNAs were predicted to have roles in biomineralisation – one of which being miR-100, which regulates growth and development in humans [26], [27], [94], [95]. Further, among the targeted transcripts, we found several transcripts which are predicted homologues of genes involved in calcium and bicarbonate ion transport that are directly regulated by miRNAs (miR-506 targets human anion exchange protein 2 [100], while miR-17 targets polycystin-2 [101]). A potential involvement of miRNAs in regulating ion transport is intriguing, given the significance of these processes in relation to ocean acidification and associated consequences to coral calcification [102]. However, future experiments (e.g in-situ hybridisations, gene expression assays, or immunoprecipitation studies) are essential in unequivocally verifying these predicted interactions.

In conclusion, our study provides strong support for the presence of a functional RNAi machinery in *S. pistillata* as highlighted by our phylogenetic analyses, the strong conservation of key RNAi protein domains, and the presence of conserved miRNAs. miRNAs seem to affect a variety of biological processes in corals, but further studies that focus on the coordinated expression of miRNAs and associated target mRNAs under different conditions, as well as their interaction with RNAi proteins, are needed in order to identify, characterise, and understand the operational miRNAome in scleractinian corals.

### Data access

All small RNA and RNASeq data are available in the NCBI SRA (Sequence Read Archive) under accessions SRR1130519 and SRR1125978 respectively. This Transcriptome Shotgun Assembly project has been deposited at DDBJ/EMBL/GenBank under the accession GARY00000000. The version described in this paper is the first version, GARY01000000. Names of the miRNAs are temporary, as miRBase (the microRNA registry) requires acceptance of manuscripts prior to assigning names to newly identified miRNAs. Other data are available as Supporting Information.

## Supporting Information

Supporting Information S1 ***Stylophora pistillata*** **transcriptome (43,493 genes/loci ≥250 bp, DDBJ/EMBL/GenBank accession GARY00000000).**
(ZIP)

Supporting Information S2 ***Stylophora pistillata*** **transcriptome BLASTX and GO annotation (43,493 genes/loci ≥250 bp).**
(ZIP)

Supporting Information S3 **Candidate RNAi proteins in** ***Stylophora pistillata*** **.**
(DOCX)

Supporting Information S4 **Alignment of sequences used to construct maximum-likelihood phylogenetic trees (FASTA format).**
(FA) Click here for additional data file.

Supporting Information S5 **Graphical alignment of the PAZ domains in Argonaute and Piwi proteins.** Of note are the strong conservation of glutamate (E) at position 137 (mutants produce insoluble protein) and phenylalanine (F) at position 72 (required for RNA binding). However, the phenylalanine at position 48 in *D. melanogaster* AGO2 (also required for RNA binding) was not conserved at all. Key residue positions are marked with red asterisks.
(EPS)

Supporting Information S6 **Graphical alignment of the Piwi domains in Argonaute and Piwi proteins.** The catalytic DDX triad, which contributes to the slicing activity of the ribonuclease (marked in red asterisks), is located at positions 46, 140 and 284 or positions 46, 140 and 155. This triad is present in one *S. pistillata* candidate, but not in two others, most likely due to the transcript sequences being incomplete.
(EPS)

Supporting Information S7 **Graphical alignment of the first RNase III domain in Dicer and Drosha proteins.** Remarkably, all of the key acidic aspartate (D) and glutamate (E) residues, which are involved in the coordination of a divalent metal cation, are conserved across the candidate homologues and known sequences.
(EPS)

Supporting Information S8 **Graphical alignment of the second RNase III domain in Dicer and Drosha proteins.** Similarly, most of the aspartate (D) and glutamate (E) residues involved in the coordination of a divalent metal cation are conserved - perfectly conserved for the Drosha candidate (“Locus_18820”), while the Dicer candidate (“Locus_10081”) only has the first two key residues. Both sequences however align well to known Dicer and Drosha proteins.
(EPS)

Supporting Information S9 **Graphical alignment of the dsRNA-binding domain in Pasha.** The key alanine/alanine pair (AA, positions 21 and 22) and alanine/serine pair (AS, positions 139 and 140) involved in the binding of dsRNA are also present in the *S. pistillata* candidate Pasha. As Pasha is an essential cofactor of Drosha, it lends support to the positive discovery of Drosha in *S. pistillata*.
(EPS)

Supporting Information S10 **Graphical alignment of the methyltransferase domain in HEN1.** The residues involved in Mg^2+^ coordination (positions 118, 121, 122 and 123) are well-conserved across the aligned sequences; residues associated with the cofactor AdoHcy and 3′ terminus (other positions marked by a red asterisk) are also well conserved.
(EPS)

Supporting Information S11 **List of additional criteria used to select** ***bona fide*** **miRNAs in** ***S. pistillata*** **from miRDeep2 results.**
(XLSX)

Supporting Information S12 **Enriched GO terms (P<0.01) associated with the set of 31** ***bona fide*** **miRNAs identified in** ***Stylophora pistillata*** **.**
(XLSX)
