# Supplementary material for: Identification of MicroRNAs in the Coral Stylophora pistillata
Source: PLoS One. 2014 Mar 21;9(3):e91101. doi: 10.1371/journal.pone.0091101 (PMC3962355; doi:10.1371/journal.pone.0091101)
Supplement: Supporting Information S3 — Candidate RNAi proteins in Stylophora pistillata . (DOCX) [file pone.0091101.s003.docx]

**Table S3:** RNAi-associated candidate transcripts in *S. pistillata*. For all candidates, the top hit from reciprocal searches against SwissProt lends strong support to the inferred functions of those proteins. All of the top hits have metazoan origins as well.

| **Protein annotation** | **Length** | **Key protein domains present (InterProScan)** | | | | | **Reverse BLAST to Swiss-Prot, top hit** | | |  |
| --- | --- | --- | --- | --- | --- | --- | --- | --- | --- | --- |
|  |  | **Paz** | **Piwi** | **RNase III** | **dsRBD** | **MTase** | **Annotated function** | **Organism** | **e value** | **Inferred function** |
| Locus_2397 | 699 | 1 | 1 |  |  |  | Piwi-like protein 1 | Mus musculus | 0 | Piwi |
| Locus_5384 | 927 | 1 | 1 |  |  |  | Piwi-like protein 2 | Danio rerio | 0 | Piwi |
| Locus_10081 | 1670 | 1 |  | 2 |  |  | Dicer 1 | Cricetulus griseus | 1.3E-127 | Dicer |
| Locus_12001 | 791 | 1 | 1 |  |  |  | Argonaute 2 | Mus musculus | 6.0E-152 | Argonaute |
| Locus_18820 | 575 |  |  | 2 |  |  | DROSHA | Homo sapiens | 0 | Drosha |
| Locus_27271 | 530 |  |  |  |  | 1 | HENMT1 | Rattus norvegicus | 2.1E-60 | HEN1 |
| Locus_42865 | 583 |  |  |  | 1 |  | DGCR8 | Mus musculus | 1.8E-89 | Pasha |

**Protein sequences**

>Locus_2397_STYPI

MTGRARGRSRGRGRGGGDGSRPGDCAQRVAPEQVPVVVGRGRSRGPPSGQISQPQRPVAPPTEAMAGMSVKEQRPPVQSVSGAREQRPRLSRDVDDNVVTKPAHLIDKMGNTGSGIDLVTNHFKLKTKTNFGVYQYTVSFNPEVEAKRARVAMLRSKSDLLGRVHAFDGMTLYLPTRLSEVETRCNITRPQGDVVQMAIKFTNEVPPDSPSMLQLYNIIFRRILKNIGMQQVGRNYYNTSQPIVIQKFGLELWPGYSTSILPYETDFLLSADVSHKVLRTTTVLEYLYELYERYERTGRKGDFHGEATKKLVGQIVLTRYNNKTYRIDDINWDLFPSNTFHGRFKGEERDITYKEYYQETYGKEIGDMEQPLLVSRLKREPGQMTRIPNGELLLVPELCFLTGLTDEMRNDFGMMKDLAVHTRVDPKARNQSLMRFINSIKSNADASAELSGWNLEFEDKTLQMSGRVLPPEKIFQKNKSFSYEPRTAEWSREMRGNPLQSTVNLNNWTLIYTNRDSGNAKDFAQTLSRVCGPMGIQVSKPYEFELNNDRTESYLHAINERYCSSLQMVVAVLTTTRKDRYDAIKKCCCLEKPVPSQVIVSRTISKKQMLMSVCTKIGIQLNCKLGGEAWAVEIPLKKTMVIGIDTYHDSLQKGRSVGGFVASTNPTLTKYYSRVTFQHTGMELIDGLKTSMTGISSCI

>Locus_5384_STYPI

MYPQQQQQPSHQQGPQYHQQPRYPSNVHQIPQQNQSQMPQQYQRASGGRGQLYGHGGPAYGGPAPPYGQQPASTTLGQQQAVSYGRGAQVATSYGRGAPAPSVPASYGRGGTTGNTPQSQPFATPTPAQPSAPSERCLSPPQQTKGPQAPVEQMAGLKVSDDIIKTQTGEKGSAGKQMSLCANFIPIRFIEQKIYQYHVSYSPEIDSKKTRHGLLKSHKEVLGPASAFDGATLFLPKQLESPTVLESERRTDGEKVTIAVTFTRVVPPDDCLQLLNIIFRRVMSRLHLTQVGRYYYDPHRPASIPQHKIELWPGYITSIQCYEGGMMLLCDVSHRLLRTETCYDLMHQLYLKRKDRFQEECKKQLVGNIVLTRYNNKTYRIDDIAFDQNPTSTFTFHTGEQMSYVDYYSKVYGIELQDLEQPLLIHRPKDKEQQKGRKLGLVCLVPELCNITGLTDAVRQDFRVMKDIAAHTRVGPMQRQQAMLKFIDNINSCPEALQELTSWGVQLDQTMLQTEGRLLPFEKIILGSTSFISSPQADWGQQAVKEQVITPVPLRNWLVLYVNRDKSKAVEFVSMMNKVTPAMGIEVHQPNMLELRDDRTETYLRMIREHLNPQTQVVVVIFPTSRDDRYSAVKKLCCVESPVPSQVINAKTISQQNKLRSVTQKIALQINCKLGGELWALDIPLKSLMVIGIDVYHDASRGGRSVCGFVASMNKSLTRWNSRVCFQSPGQELIDGLKMCLVSSLKKYHEINHTLPDRIVVYRDGVGDGQLKTVAGYEVQQLSECFVHFGVNYHPKMAVVIVSKRINARIFAAQGGGPRPKLDNPGPGTVLDHTITRKDWYDFFLISQHVRQGTVTPTRYIIVHDKSELKPDYMQRLSYKLTHLYYNWPGTVRVPAPCQYAHKLAYLVGQNIHNEPSHDLSDRLFFL

>Locus_10081_STYPI

MERAQEMSFVWPHHSVDIAERVLASEQSCVVCVSRDKSKTFIAFNVCQEFWRRNVKCGKMILLVDDEKATIVEPFLTGISNQSLLSVKAFFKKPGVEDEWNCSFSMCDIIVTTGEAFAQRLIARTVELSLCSLIIFDQCHLALNQEHHFAIILQKMKESNLESRPHIMGLSSQILCHQDCSEDMEIFLTSLEQIFNCRTLISSDLLALNRYGEYTEMEIGCFNCSHVQDPHIFNLSKIFERALFFLKDYRMNQVTETCVIFARHILTEGHRVLLLLGSWCTWYMAKITVREIDKLEKKSVDGEIVLILQFCRTQIRLVISILEQEKLEVSVNDLTDLGSILLSRTSMHLKPEHQEVNNSVPTLGTNQGLYEEPLQSCESCLIDKVSSSESQDILQGPNTQSLNLKTSYGTKNQCNDPLCVILVPSTIIAKALNSLLNKLSNAVVKYSFLKSACIHGNKAKEEMKESSFSDEVDENVMVCVQDGSVNVLIATFEIEQELYAKRCSLLIRLGMPNAYENYYSVKSKLKSAGGSLVVLVREEELAETEAKYQIFQRIEKLLWERCPNSRPPGHETRKLFQESQADVYQPLGEGGAKVELLSSIALVNSYCGKFPSSGVGYPVPVCRMQEVWSNKEVQFVATLFLPRNSPVYQPIKGSVTLKKTDINEDTMRKAKVKSEMLAALEACRLLHKREELTDDLQPLFRWHAYNLTVQHEPCDCEVADGGVGVDGTVTRNRAYKRKFPTLLENNFPVCGEPCYVYSLTMELTKPWNFERALRSRSKAKATSDSYSIGVLSRKPLPKLPRIAIFDRAGEVTVTVTECCPQSIFLTADQLSLLQRFTEYVFKEIARPKKESSATFLSFDPTIAFSGYYILFLKDASSSRSLAGLSSNNHKEVAFDFMFSLEDKLGCFKNPVYSGPPDITDAEIFRDTVVTATYNEKRSHYYVADICYDLSPSDPFPNIEVAGTFAEYVKIRYDVEVSLDQPMLDVDHTSSRLNFLLPKYENFKGQHPRIPEKNSKRSRKSKVYLIPELCSIHPVPGHLWRQLSNLPAVLYRIESLLVAEELRCWVVRDLGIGVVDWPKDVPLPPVTVGETMGEEILPAIGSSAVESNGKRSSQVALVNLTSPFSAQLPEVFMDLKISNQVELTEKSAAKSLAGGLSNGARESCLSMSSENFLVEHSCAETECKPLFRPAPHQGLLIPSKEKEDFWPNKLSDLNVPLPTERRAFDCDSIPANDKESEVSAVSIWTDPLLSNLYRTCGPPSSLILRALTTTSAGDVFSLERLEVLGDSFVKYAISSSVFFKHRYENEAVLSFLRGIKVRNRQLFYLARQRGLPSYMFTRMFNPLVNWLPPGFYLDDDSNAENSGYEHRRLISDTFLDEVDDEEDDESIFAETESSGYNSDCRQGESPDIQLNSYLHVCCSDKSIADCTEALIGAFLLCFGSDGAFKFLEWLGMEIERDEKDENLVHKPSSDDSAGDAVPKPASTMCHHDTSHVPQQASETLQENLSLLDDETVPSSSIDGLSSEHESTVDEFKDVEEALHYRFHDKSLLMQAFTHSSLPGDYNSVRNSYEQLEFLGDALLDFLVMRYLYVNHRHMSPGELTDLRTALVNNYSFAVLAVKLGFPRHLRSCSPQLFGMVNKFMVKLKEKERKHAITHRKNDEVRLSRLTKTLVC

>Locus_12001_STYPI

MSSQGQGKNKRKGRGRGNAGRGRGRGHDLPPTPGTTDRVGKPLEKSGITIEEKPDYLNPKDSSTADFDEKLAAGACGNDLLNDQKDTVRPEKSSTTASRATTTVEEHNQTGRSSVETPSKSRKIKGRQRDIPASSSDDKGGGAPAADASSIKSVGATTPSSAAATSSTVAVNKEVENGTKKLGSSLGSGGHTTHQTQGNGGPHPPKRPGHGTKGHPIALRANFFRLNISPELSDLYHYDVEITPDKCPRSDKRDVVNKIIEEYKHTTFQGHHPAFDGAKNLYSRIKLPVPAELVVKLPGKDGGKERNFKVKIQFAATVSLLELNNFLSGKQNGKIPQDAVQALDIVVRQMPSLYYTPVGRSFFPLDGRRSPLGAGCEVKFGFYSSIRHSEWKAMLVNIDVSAKGFDKEQAFVPDFLCETLGVRAHNIEDRSFQPASWKLEKAIRGIRIQTTHAAPIKRKYTVWGFSGESAERMQFDVTDEGTGRTYKTTIAEYFRDRYGLTLRYPHLPCLLVGPKKDRYLPMEVCTIIPCHRKHLSEQQTANMIRSTARPAPERQSNIQHWAQRVTQASGKYLRDEFHTSISTEMVKVEGRVLPAPTINLGPQDRPLVPLRGSWDMRDKSLHQGARINKWALACFDGRCHKDQLENFSKHMADVSSRQGLRMSEQPVVVAYDRGIRDVESLFSKWVVDFPGLQLIMAVLPERDKEIYPELKRVGDNVIGIPTQCVQSKHVHRINLQVCANIGLKINSKLGGINHDIDPGVKSPVFREPVIIFGADVTHPSPTENGIPSIAA

>Locus_18820_STYPI

MRVKSAMRRDVLVEVSSKGMIKTGLKSDVCQHALLLPVFVHHVRYHMCLKTLDERMGYVFKDRSLLQLALTHPSYHLNFGMNPDHARNSLSNCGVKQPRYGDRKIRHLHTRKKGITQLIRVMSNLGKMEEHQSMIRHNERLEFLGDAVLELISSVHLYFMLPSKTEGGLAMYRSALVQNKHLAQLAKKLGLSDYMQYSHGPDLCVEDDLNHAMANCFEALLGAMYLESGLTSATILFTNVAFEEQELRDVWTNLPKHPLQAQQPDGDRHLIASSPILQKLEEFEKASGIEFTHIRLLARAFTHPQVGFNNLTLGSNQRMEFLGDSVLQFVVSVYLFKHFPEHHEGHLTLLRSSLVNHRIQATIARELGLDKLINFGNQGMNSYREKLLADILEAFVAALYVDKGLRYVETFCHVCLFPRLEDFIINQDWMDAKSQLQQCCLTSREQGKIPDLPQYKVLQNRGPAHHKRYTVAVYYKGRRMGSGEGKSIQQAEMAAAKDALASHYFPELARQKRLLDQKHQGRRRNYWNKVPRKESEEKRDREEDVEVLPRWEEKETFEEDDNTDVTQEECQPQEQ

>Locus_27271_STYPI

MGDTANDTTIIGHVARGDKPLLSTENEGSFKGPKFDPPVYRQRYAAVCELVKERQAKKVLDFGCAEAKLVKTLISQDNLIHLEEVVGVDIDQQLLEESKFRIKPFTADYLRPRSHPFKVSLYQGSIAEADERFIDFDIVACIELIEHLEPDILDLMPKAVFGQLSPKVVMVTTPNVEFNVLFPDLKGFRHYDHKFEWTRAEFEKWSNTQALKYNYTVRFDGIAHGPKGTEHLGCCSQMAVFERMSSFSASRKESGIGQPYNLIAEVQFPFKKDTRTEEEKILQEVEYILWMLSSQEEVHDSEESDVDPLSSDKDETSCHYDQNHLMHDEDCAKGGNDEAQRHVYLLKELLGFRSLQKFCNEIEKLRSVLKGSSRFCLTEDERGVLWQHQSSSQWSSEESDTGWDVCGDEQEVYESKRPDSAWTEPENWDAADEITTDANVKQTEANTEVCWDSANGCNNKCWNGEDWSKLEEYGADDEFQEENSFNFHNSYTVAVPLESDGSGSDDIDGNEIGDLVWITENTAITDANPK

>Locus_42865_STYPI

MSVDISVNELAVVVKAPTVLTVGENSELRTEIEKMENTMEKVSSSAGMDIPTYSKEESTDEEMPFETERIPLKRKSEEDLLDHNHKRRMTEGDQADDWENVISTTGDEKQEGENPTDEQERKVIILKGNLPKLALPEGWIALNHRSGGIIYLHKPSRVCTWSRPYHIGGGSVRKHDVPLAAIPCLHQKKGINQENTEETKSENGTSKSKNPAGSILNALNTQDVEKCVANTSVVKNTSEEQEETTDSNKNTPPTLELLDIGELGSYLSTIWEFQTLTSEQERYAVMALPQTEDDVELPSSLECLSYSVKGAENGKSPSKYCLLNAGGKTPVAILHEYCQRILKSKPVYLASESASSDSPFVAEVQIDGIKYGSGTGSSKKIARQIAAESTLEVLLPGMFKKIRDYQISDAELEFFDQVDILNPRLFEFCSKTSLPSPSQILEECLRRNQGICSSIEFKTVCGQNKSLSFIITCGKHTATGPCKNKRNGKQLASQHILKKLHPHLEKWGALIRIYCDRPTGSIKKYKKDDSETANEKSGGNSSTNTDLLERLKSEMRKMYSENENANRMDAMDKPAEPVFTVTI
